# Moving from supported to independent living: what are the barriers and facilitators for individuals with psychosis?

**DOI:** 10.1007/s00127-023-02586-x

**Published:** 2024-01-08

**Authors:** Anika Poppe, Natalia Tiles-Sar, Stefan R. A. Konings, Tesfa Dejenie Habtewold, GROUP Investigators, Behrooz Z. Alizadeh, Therese van Amelsvoort, Agna A. Bartels-Velthuis, Richard Bruggeman, Wiepke Cahn, Lieuwe de Haan, Frederike Schirmbeck, Claudia J. P. Simons, Jim van Os, Richard Bruggeman, Behrooz Z. Alizadeh, Lisette van der Meer

**Affiliations:** 1 Department of Clinical and Developmental Neuropsychology, University of Groningen, Grote Kruisstraat 2/1, 9712 TS Groningen, The Netherlands; 2 Department of Rehabilitation, Lentis Psychiatric Institute, Zuidlaren, The Netherlands; 3 Department of Epidemiology, University Medical Center Groningen, University of Groningen, Groningen, The Netherlands; 4 Department of Psychiatry, Rob Giel Research Center, University Medical Center Groningen, University Center for Psychiatry, University of Groningen, Groningen, The Netherlands; 5 Department of Psychiatry, Interdisciplinary Center Psychopathology and Emotion Regulation (ICPE), University Medical Center Groningen, University of Groningen, Groningen, The Netherlands

**Keywords:** Sheltered housing, Supported housing, Functional recovery, Schizophrenia, Markov chain modeling

## Abstract

**Purpose:**

Living independently, as opposed to in sheltered housing or with caregivers, is an important aim in the recovery of individuals with psychosis, but the transition to independence can be challenging. This study aims to investigate how individuals with psychosis move between living arrangements and to identify the barriers and facilitators of moving towards independence.

**Methods:**

The living arrangements of 1119 individuals with non-affective psychosis from the Genetic Risk and Outcome of Psychosis study were assessed at baseline, at three- and six-year follow-ups and further categorized as either supported (sheltered housing or with parents) or independent (single or with partner/family). We estimated the probabilities of transitioning between the living statuses and investigated the influence of demographic characteristics, symptomatology, cognition, social support, and premorbid social adjustment on transition using Markov chain modelling.

**Results:**

The majority of individuals living in supported housing remained there during the six-year follow-up period (~ 60%). The likelihood of moving from supported to independent living was twice as high for participants who were younger, five-to-six times higher for women, twice as high for individuals with better overall cognition, and five times higher for those with a course of low positive symptoms.

**Conclusion:**

This study highlights that a large group of individuals with psychosis in supported housing is unlikely to move to independent living. Older men with cognitive impairments and who show continuous severe positive symptoms are the least likely to move living independently. Tailored interventions for these at-risk individuals could increase their chances of moving to independent living.

**Supplementary Information:**

The online version contains supplementary material available at 10.1007/s00127-023-02586-x.

## Introduction

In the last few decades, the large-scale deinstitutionalization of psychiatric treatment has been executed worldwide [1]. While individuals with psychosis used to receive long-term inpatient treatment in psychiatric institutions, now they are often offered community care while living in their own homes or sheltered housing. Clinicians, scientists, and individuals with psychosis consider this change a significant step forward regarding independence and integration into society [2], but many people with psychosis who live in supported or sheltered housing would prefer to have their own home [3, 4]. The experience of moving into their place is an important turning point in the recovery process, representing a sense of freedom, space, and privacy [5]. Despite this desire to live independently, not all individuals with psychosis move to independent housing. Therefore, there is an evident necessity to understand the underlying factors that hamper or facilitate this transition to pave the way towards independent living for more people.

Though many studies have investigated predictors of daily functioning in people with psychosis [6], there is little published data on predictors with independent living as a separate outcome. Cognitive functioning has been identified as a predictor of independent living, but the results are controversial. A small study of 100 individuals with schizophrenia found that neurocognitive and social cognitive measures were predictive of living independently one year later [7]. Another study found that shorter illness duration and available social support at baseline were associated with being discharged from residential facilities in Italy, but neurocognitive measures were not related to that outcome [8]. Elsewhere, individuals with fewer negative symptoms [9–11], better cognitive functioning [10–12], better premorbid social adjustment (school and social performance) [13], available social support [8], and shorter illness duration [8, 10] have been identified as more likely to live independently. On the other hand, having fewer positive symptoms is not associated with a more independent living situation [10, 14]. Although symptoms change over time and their development differs across individuals with psychosis [15], previous studies measured the factors associated with independent living at a single time point rather than longitudinally. Notably, most studies investigate associations between predictors and housing arrangements rather than predictors of transitioning, for example, moving from a supported to an independent housing arrangement.

### Objectives

Firstly, this study investigates how individuals with psychosis in the Netherlands and Belgium move between four housing arrangements: single, with partner/own family, with parents, or sheltered housing. Secondly, we study the relationship between cognitive functioning, positive and negative symptoms, premorbid social adjustment with transitions from supported (i.e., sheltered housing or with parents) to independent (i.e., single or with partner/own family) living and vice versa. Considering longitudinal clinical variability among participants, we speculate that more favorable courses of positive symptoms, negative symptoms, cognitive functioning, and premorbid social adjustment predict a transition from supported to independent living arrangements.

## Methods

### Study design

We analyzed data from the Genetic Risk and Outcome in Psychosis cohort study (GROUP, data release 8.0) that recruited 1119 individuals in the Netherlands and Belgium diagnosed with a psychotic disorder. The inclusion criteria were being 16–50 years, diagnosed with non-affective psychosis as per the Diagnostic and Statistical Manual of Mental Disorders (fourth edition; DSM-IV), good command of the Dutch language, and being able and willing to give written informed consent. Between April 2004 and December 2013, participants were assessed at baseline, after three years, and after six years. The assessments were conducted by a trained research assistant, psychologist, psychiatrist, nurse, or Ph.D. student. The study procedures have been described in detail elsewhere [16].

### Outcomes

The current housing arrangement was assessed at each assessment point. Participants were asked to indicate their current living situation: “Single”; “With parent(s)”; “With partner/family”; “Sheltered living”; or “Other”. Due to the lack of available information regarding the “other” category, our understanding of the living situation of individuals falling into this category remains unknown. Hence, we have chosen to exclude this category from our analyses (baseline: *n* = 68; 3-year follow-up: *n* = 38; 6-year follow-up: *n* = 24). For the association analysis, we grouped “Single” and “With partner/family” into “Independent living” and “With parent(s)” or “Sheltered living” into “Supported living” to simplify the interpretation and gain more power.

### Predictors

#### Baseline predictors

Cognitive functioning was assessed with a neuropsychological test battery comprising ten measures in the domains of processing speed (Digit Symbol Substitution, total score); attention/vigilance (Continuous Performance Test: reaction time and sensitivity score); acquired knowledge (Wechsler Adult Intelligence Scale (WAIS)-III Information: total score); working memory (WAIS-III Arithmetic: total score); verbal learning and memory (15-word learning task: total correct at immediate and delayed recall); executive functioning (WAIS-III Block Design: total score, Response Shifting Task: reaction time cost and accuracy); and social cognition (Degraded Facial Recognition Task: total correct, Hinting Task: total score and sensitivity). The test battery has been described in detail elsewhere [17]. All test results were standardized and coded so that a higher score indicates a better outcome. We analyzed general and domain-specific cognitive functioning. For overall cognitive functioning, which included all tests, and the cognitive domains assessed with more than one test, we calculated composite scores by averaging the z-scores of all corresponding tests.

Symptom severity over the past week was assessed with the Positive and Negative Syndrome Scale (PANSS; 18). We used the PANSS subscales for positive and negative symptoms. Additionally, we calculated the two subscales for negative symptoms as recommended by van der Meer et al. [19], namely expressive deficits and social amotivation [20]. Premorbid social adjustment (i.e., social life and school performance) was assessed at baseline with the Premorbid Adjustment Scale (PAS; 21), a retrospective measure of adjustment before illness onset over three life periods (childhood, early adolescence, and late adolescence). We used an overall score representing the average of all three periods. The PANSS and PAS scores were standardized and coded so that a higher score indicates a better outcome.

Sociodemographic characteristics were collected at baseline and standardized. These were sex (male/female), age, and IQ (WAIS-III). Additional clinical parameters were also measured, such as illness duration (years since first psychotic episode), general functioning (Global Assessment of Functioning, disability subscale), and social support by the number of unmet needs (Camberwell Assessment of Need 22).

#### Trajectories

We have previously applied group-based trajectory modeling to identify different courses (or trajectories) of cognitive impairment based on composite cognitive score [23], positive and negative symptoms based on PANSS subdomains [24], and premorbid social adjustment based on overall PAS score [25] As a result, participants with similar courses were grouped for each domain. In the current study, we used the following trajectories as predictors of housing state transition:

- Cognitive impairment: constantly (1) none, (2) mild, and (3) moderate-to-severe;
- Positive symptoms: constantly (1) low, (2) moderate, and (3) severe;
- Negative symptoms: (1) low, (2) high-decreasing, and (3) high-increasing;
- Premorbid social adjustment impairment: (1) none-to-mild, slow decline; (2) none-to-mild, rapid decline; and (3) moderate-to-severe, slow decline.

### Statistical analysis

Baseline differences of predictors by housing arrangement were examined by χ^2^-test or *t-*tests with Bonferroni correction, as appropriate.

We used multi-state modeling to explore transitions between different housing arrangements over six years and to identify the factors that predict these transitions. Firstly, we explored transition probabilities based on observed frequencies based on the following formula $P\left(  S0 T0 \to S1 T1 \right) = n\left(  S0 T0 \to  S1  T1 \right)/n\left( S0  T0 \right)$, where:

- $$P$$$$\left(  S0 T0 \to S1 T1 \right)$$ is the transition probability from state S_0_ at time T_0_ to state S_1_ at time T_1_,
- $n\left( S0 T0 \to S1 T1 \right)$ is the number of individuals who transitioned from state S_0_ at time T_0_ to state S_1_ at time T_1_,
- $$n($$$$ S0 T0)$$ is the total number of individuals in state S0 at time T0 who made any transition.

Secondly, we used Markov chain modeling to understand the impact of different factors on theses transitions. Markov chain modeling is a method for modeling transitions under the basic assumption that the distribution of the future state depends only on the current state and not on previous states. In our case, an individual’s future housing arrangement should only depend on their current housing arrangement. We checked this assumption utilizing logistic regression and testing whether the housing at the six-year follow-up is independent of the baseline given the state at the three-year follow-up. The chain can be time-homogeneous, meaning that the transition probabilities do not change with time. We tested the time-homogeneity assumption by including time of assessment as a covariate. The analyses were conducted using the ‘msm’-package (version 1.6.8) [26] implemented in the statistical environment R (version 4.0.3) [27].

The associations of transitions with predictors were determined as hazard ratios (HRs). As we standardized the continuous predictors, the HRs for the continuous variables represent the ratio of the transition probabilities with one standard deviation difference determined separately for each transition. The reference group is the transition probability when standardized covariables are set to 0. For the categorical variables, the HR is the ratio of the transition probability in one group compared to the reference group (e.g., the best functioning group). The results are presented by maximum likelihood estimates and 95% confidence intervals, computed by assuming the normality of the log-effect. The associations were tested in univariate analyses followed by multivariate analysis with all significant predictors. For additional information on model fit, we report the Akaike information criterion (AIC), where the smaller value corresponds to a better fit.

## Results

**Fig. 1:**
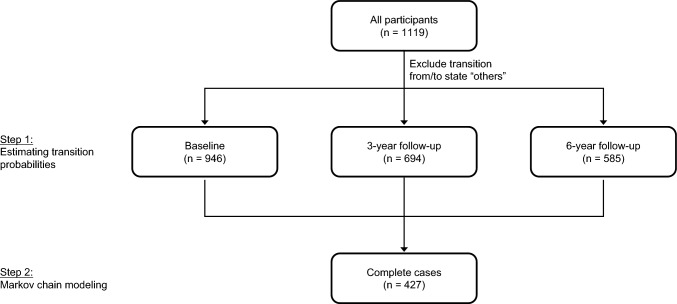
Flowchart of cases included in analyses. Note: The transition probabilities were estimated from observed probabilities of transitioning (i.e., including only cases with information on housing arrangements at least from baseline to 3-year follow-up or 3-year follow-up to 6-year follow-up). For the Markov modeling only complete cases (i.e., information on housing arrangement at all three time points) were included

**Fig. 2:**
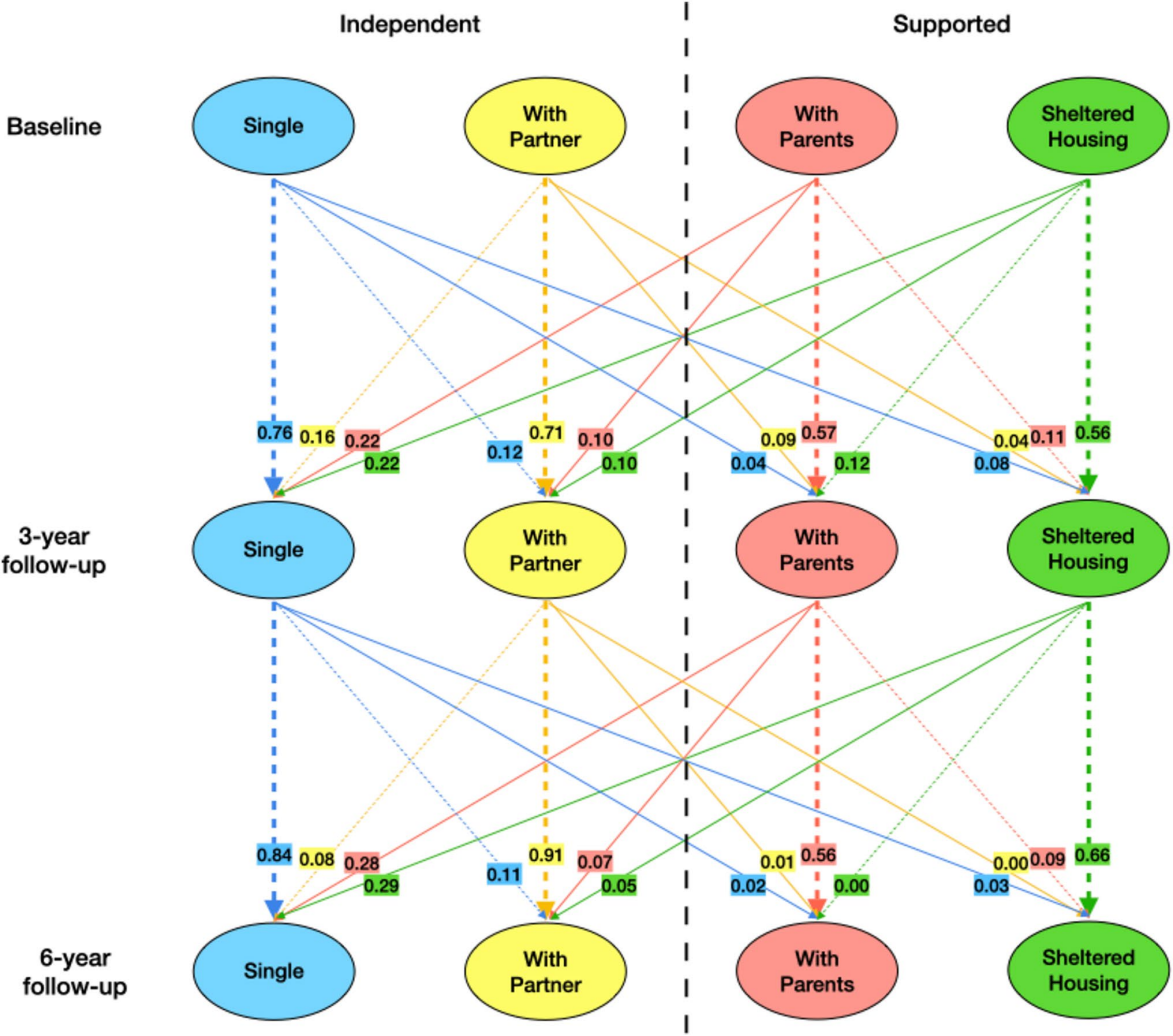
Observed transition probabilities of moving across living arrangements over six years follow-up. Note: Dashed lines represent transitioning within supported or independent housing. Solid lines represent transitioning from supported to independent living or vice versa. The colors represent the housing arrangement someone is transitioning from (blue = single; yellow = with partner; red = with parents; green = sheltered)

Figure 1 gives an overview of the cases included for each analysis (see Table S1 in the supplementary material for the descriptive characteristics of the complete sample, the completers and those lost-to follow-up). Based on observed probabilities (baseline: *n* = 946; 3-year follow-up: *n* = 694; 6-year follow-up: *n* = 585) of transitioning between housing states (Fig. 2), most people remained in the same housing arrangement, with the highest number staying single or with a partner. Among those living with parents or in sheltered housing at baseline, approximately 60% remained in the same living arrangement (measured at each follow-up). When transitions occurred, people mostly moved from living with parents or in sheltered housing to living alone. In general, fewer transitions were observed during the second follow-up period, while the probability of staying in the same housing arrangement increased for all categories except living with parents.

Only complete cases could be used for further analysis, so 692 of the 1,119 participants were excluded because of missing housing data at one or multiple assessment time points (no information: *n* = 23; two missing values: *n* = 294; one missing value: *n* = 325). Thus, 427 participants were included in the predictor analyses with Markov chain modeling. Table 1 presents the descriptive characteristics of the complete cases by living arrangement. For the merged categories (i.e., supported and independent living), there were significant differences (*P* < 0.05) for age, illness duration, general functioning, premorbid social adjustment, and trajectory of premorbid social adjustment. ‘

**Table 1:** Baseline characteristics of the complete cases across living arrangements (before standardization)

	Independent living			Supported living		
	Single	With partner	Merged group	With parents	Sheltered	Merged group
	*n* (%) / mean (SD)	*n* (%) / mean (SD)	*n* (%) / mean (SD)	*n* (%) / mean (SD)	*n* (%) / mean (SD)	*n* (%) / mean (SD)
Sex*						
Male	133 (80%)	23 (48%)	156 (73%)	133 (78%)	32 (76%)	165 (77%)
Female	33 (20%)	25 (52%)	58 (27%)	38 (22%)	10 (24%)	48 (23%)
Age**	30.4 (6.3)	35.1 (8.6)	31.4 (7.1)	23.5 (5.1)	26.7 (5.7)	24.1 (5.4)
Illness duration (in years)**	6.3 (5.3)	7.0 (6.9)	6.4 (5.7)	3.8 (2.9)	7.2 (5)	4.5 (3.7)
IQ	98.9 (16.2)	98.3 (18.8)	98.8 (16.8)	96.8 (15.3)	92.3 (11.5)	95.9 (14.7)
PANSS negative symptoms	– 14.7 (6.2)	– 12.4 (4.9)	– 14.2 (6.0)	– 15.0 (6.4)	– 14.6 (7.1)	– 14.9 (6.5)
PANSS positive symptoms*	– 13.1 (6.1)	– 11.3 (4.9)	– 12.7 (5.9)	– 12.2 (5.5)	– 16.4 (7)	– 13.0 (6)
Cognitive composite score (standardized)	0.1 (0.5)	0 (0.7)	0.1 (0.6)	0.1 (0.5)	0 (0.5)	0 (0.5)
Premorbid social adjustment**	– 1.9 (0.9)	– 1.4 (0.6)	– 1.8 (0.8)	– 2.0 (0.8)	– 2.3 (1)	– 2.1 (0.9)
GAF general functioning**	56.7 (15.5)	65.9 (15.0)	58.7 (15.8)	55.0 (14.9)	51.9 (15.4)	54.4 (15.0)
Unmet needs of social support	2.9 (2.5)	1.9 (2.3)	2.7 (2.5)	3.0 (2.9)	3.2 (2.6)	3.1 (2.9)
Positive symptoms trajectories*						
Low	119 (72%)	41 (85%)	160 (75%)	131 (77%)	18 (43%)	149 (70%)
Moderate	39 (23%)	6 (13%)	45 (21%)	31 (18%)	17 (41%)	48 (23%)
Severe	8 (5%)	1 (2%)	9 (4%)	9 (5%)	7 (17%)	16 (8%)
*Negative symptoms trajectories*						
Low	119 (72%)	41(85%)	160 (75%)	129 (75%)	30 (71%)	159 (75%)
High, decreasing	25 (15%)	5 (10%)	30 (14%)	17 (10%)	8 (19%)	25 (12%)
High, increasing	22 (13%)	2 (4%)	24 (11%)	25 (15%)	4 (10%)	29 (14%)
*Cognitive impairments trajectories*						
No	82 (49%)	23 (48%)	105 (49%)	93 (54%)	14 (33%)	107 (50%)
Mild	63 (38%)	18 (38%)	81 (38%)	54 (32%)	19 (45%)	73 (34%)
Moderate-to-severe	21 (13%)	7 (15%)	28 (13%)	24 (14%)	9 (21%)	33 (16%)
Premorbid adjustment impairment trajectories**						
No-to-mild, slow decline	116 (70%)	42 (88%)	158 (74%)	102 (60%)	19 (45%)	121 (57%)
No-to-mild, rapid decline	12 (7%)	2 (4%)	14 (7%)	28 (16%)	7 (17%)	35 (16%)
Moderate-to-severe, slow decline	38 (23%)	4 (8%)	42 (20%)	41 (24%)	16 (38%)	57 (27%)

Note: *GAF* global assessment of functioning, *PANSS* positive and negative syndrome scale

*Significant differences between groups for initial housing categories (*P* < .05)

**Significant differences between groups for initial and merged housing categories (*P* < .05)

### Markov chain modeling

Logistic regression showed that the Markov assumption of independence was not violated. The housing arrangement at six years was not significantly associated with the housing arrangement at baseline (*P* = 0.56), given the housing arrangement at the three-year follow-up.

Based on modeled transitions, the estimated probability of staying living independently after three years was 0.92 (95% CI 0.89–0.94), while moving to supported living was 0.08 (95% CI 0.06–0.11). The probability of staying in supported housing was 0.66 (95% CI 0.61–0.71) and moving to independent housing was 0.34 (95% CI 0.29–0.38). Modeled for ten years, the estimated probability of staying in independent housing was 0.85 (95% CI 0.80–0.89); moving to supported housing was 0.15 (95% CI 0.11–0.20); staying in supported housing was 0.32 (95% CI 0.27–0.37); and moving to independent housing was 0.68 (95% CI 0.63–0.73).

As we expected, the transition probabilities changed with time, rejecting the null hypothesis of no difference in transition probabilities at *P* = 0.009. As baseline age might explain the transitional differences across the follow-up periods, we adjusted for this factor and compared (a) the time-inhomogeneous model with the time-inhomogeneous age-adjusted model for which a likelihood ratio test showed significant improvement after adjustment (*P* < 0.001) and (b) the null age-adjusted model with the age-adjusted time-inhomogeneous model which showed no significant improvement after adjusting for time-inhomogeneity in the age-adjusted model (*P* = 0.59). We concluded that time-inhomogeneity could be explained by age and, therefore, used the time-homogeneous model with adjustment for age.

### Associations with predictors

The results from the univariate, age-adjusted models are presented in Table 2. Sex, composite cognitive score, executive functioning, and positive symptom trajectories predicted the transition from supported to independent living. Based on the significant predictors and the predictors with suggestive trend towards significant association (processing speed, PANSS positive symptoms and social amotivation) from the univariate models, we developed four multivariate models. The models included, due to collinearity, either PANSS positive symptoms score or positive symptom trajectory and either composite cognitive score or executive functioning + processing speed (Table 3). Age, sex, composite cognitive score, and positive symptom trajectory were consistently predictive across the models. Aside from age, all predictors influenced the transition to independent living but not vice versa. Being female, better cognitive functioning, and a less severe symptom trajectory highly increased an individual’s chances of moving to independent housing. Older age decreased the likelihood of transitioning between housing arrangements.

**Table 2:** Predictors of transitioning between living arrangements in age-adjusted models

Predictors	Independent →Supported HR (95% CI)	Supported → Independent HR (95% CI)	AIC
Sex: female	2.95 (0.92–9.44)	**3.24 (1.20–8.73)**	434.98
Illness duration	0.61 (0.33–1.15)	0.74 (0.51–1.07)	421.56
IQ	0.80 (0.50–1.28)	1.18 (0.91–1.52)	420.40
Premorbid social adjustment	0.83 (0.53–1.31)	1.10 (0.86–1.40)	415.29
GAF general functioning	0.83 (0.52–1.34)	1.17 (0.92–1.49)	414.03
Unmet needs of social support	0.81 (0.50–1.32)	0.91 (0.71–1.17)	412.91
Cognitive composite	0.76 (0.35–1.67)	**1.73 (1.03–2.91)**	433.04
Executive functioning composite	0.90 (0.46–1.78)	**1.58 (1.03–2.42)**	431.39
Social cognition composite	0.85 (0.41–1.76)	1.09 (0.71–1.68)	438.10
Attention composite	0.96 (0.59–1.58)	0.96 (0.74–1.25)	414.84
Memory composite	0.97 (0.54–1.73)	1.16 (0.89–1.52)	429.67
Working memory	0.79 (0.50–1.25)	1.05 (0.80–1.38)	431.13
Processing speed	0.84 (0.54–1.30)	1.31 (0.99–1.73)*	425.05
Verbal comprehension	0.91 (0.57–1.47)	1.17 (0.89–1.55)	432.48
Visual perception	0.90 (0.61–1.35)	1.14 (0.89–1.46)	421.57
PANSS positive symptoms	1.28 (0.72–2.29)	1.35 (0.99–1.83)*	412.55
PANSS negative symptoms	0.95 (0.58–1.53)	1.16 (0.91–1.49)	410.56
PANSS expressive deficit	0.77 (0.50–1.20)	1.11 (0.86–1.42)	416.33
PANSS social amotivation	1.18 (0.71–1.94)	1.30 (1.00–1.70)*	414.20
*Positive symptoms trajectories*			434.68
Moderate	1.18 (0.40–3.45)	0.81(0.45–1.46)	
Severe	0.80 (0.10–6.23)	**0.14 (0.03–0.60)**	
*Negative symptoms trajectories*			440.73
High, decreasing	0.54 (0.12–2.57)	0.67 (0.31–1.40)	
High, increasing	2.05 (0.67–6.30)	0.63 (0.28–1.40)	
*Cognitive trajectories*			439.54
Mild	1.15 (0.40–3.28)	0.72 (0.41–1.27)	
Moderate to severe	2.25 (0.69–7.35)	0.60 (0.28–1.28)	
*Premorbid social adjustment trajectories*			441.01
Normal to mild, rapid decrease	1.38e-06 (1.92e-169,9.94e + 158)	0.61 (0.33–1.14)	
Moderate to severe, slow decrease	1.69 (0.62–4.58)	0.85 (0.46–1.58)	

*AIC*  Akaike information criterion. Bold numbers indicate significant differences in probabilities of transitioning (95% confidence interval of HR should not include 1). Borderline significant predictors with suggestive trend toward significance are marked with *

**Table 3:** Predictors of transitioning between living arrangements in multivariate models

Predictor	Model 1		Model 2		Model 3		Model 4	
	IL to SL	SL to IL	IL to SL	SL to IL	IL to SL	SL to IL	IL to SL	SL to IL
AIC	394.42		402.52		388.92		395.59	
Age	**0.28 (0.12–0.65)**	0.53 (0.28–1.00)	**0.32 (0.13–0.77)**	0.59 (0.31–1.13)	**0.26 (0.13–0.55)**	**0.53 (0.31–0.93)**	**0.29 (0.14–0.63)**	0.59 (0.33–1.07)
Sex: female	4.08 (0.79–21.08)	**6.00 (1.24–29.03)**	4.32 (0.95–19.61)	**5.71 (1.24–26.20)**	3.78 (0.87–16.38)	**5.10 (1.32–19.61)**	4.26 (0.94–19.30)	**5.21 (1.22–22.34)**
Cognitive composite	1.25 (0.46–3.40)	**2.44 (1.15–5.17)**	1.27 (0.49–3.31)	**2.41 (1.18–4.93)**				
Executive functioning					1.01 (0.45–2.29)	1.85 (0.98–3.49)	1.06 (0.48–2.33)	**1.90 (1.03–3.52)**
Processing speed					0.84 (0.50–1.40)	1.14 (0.76–1.69)	0.81 (0.47–1.38)	1.11 (0.74–1.68)
Positive symptoms	1.32 (0.80–2.20)	1.34 (0.93–1.93)			1.32 (0.77–2.26)	1.32 (0.89–1.97)		
Social amotivation	1.15 (0.68–1.94)	1.15 (0.81–1.65)	1.08 (0.66–1.79)	1.10 (0.78–1.55)	1.16 (0.69–1.96)	1.19 (0.83–1.71)	1.14 (0.70–1.86)	1.14 (0.81–1.61)
*Positive symptoms trajectories*								
Moderate			0.87 (0.28–2.72)	0.79 (0.39–1.61)			0.91 (0.29–2.86)	0.75 (0.35–1.61)
Severe			1.04 (0.14–7.88)	**0.20 (0.04–0.98)**			1.05 (0.13–8.22)	**0.20 (0.04–0.94)**

Note. *AIC*  Akaike information criterion, *SL*  supported living, *IL*  independent living. The results are presented as HR (95% CI). Bold numbers indicate significant differences in probabilities of transitioning (95% confidence interval of HR should not include 1). The different models include different combinations of predictors: Model 1: age + sex + cognitive composite score + PANSS positive symptoms + social amotivation; Model 2: age + sex + cognitive composite score + positive symptoms trajectories + social amotivation; Model 3: age + sex + executive functioning + processing speed + PANSS positive symptoms + social amotivation; Model 4: age + sex + executive functioning + processing speed + positive symptoms trajectories + social amotivation

## Discussion

This study describes how individuals with psychosis move between different living arrangements across a six-year period and identifies predictors of transition towards independent living. Our findings show that most individuals with psychosis stayed in the same housing arrangement for six years. Four factors predicted a transition from supported to independent housing: younger age, being female, better overall cognition at baseline, and a less severe course of positive symptoms. Notably, we did not identify any predictors aside from age that increase or decrease the likelihood of transitioning from an independent to a supported housing arrangement.

The rates of transitioning from supported to independent living were found to be low. During each follow-up, over half of the participants continued to live in supported housing, and our projections suggest that after ten years, about 32% of them would still be in supported housing. While no other studies have specifically examined moving between different living arrangements, a Dutch cohort study [28] did look at yearly transition rates between recovery states. Similar to our findings, the study revealed that 77% of individuals with schizophrenia spectrum disorders in the least recovered state, of whom 92% were partly or fully dependent in their daily living and self-care, were likely to remain in the same recovery state the following year. This suggests that the recovery process for schizophrenia is lengthy and often involves ongoing support at severe stages. A qualitative study [29] further demonstrated that individuals living in sheltered housing viewed supported living as a crucial step in their ongoing recovery process. They highlighted empowering aspects such as readily available staff support, a sense of community, and engaging daily activities. Therefore, living in a supportive environment for an extended period may be considered necessary within the context of a slow recovery process. However, it’s essential to note that ultimately, individuals with mental health issues expressed a preference for living in their own houses rather than communal residential facilities [3–5].

Participants with better overall cognition and executive functioning were more likely to move from supported to independent living. These results are in line with one previous study [7] but are inconsistent with three others [8, 9, 30]. These contradictory findings may be explained by the small sample sizes [9, 30] and older participants [8]. Generally, cognitive functioning is known to have strong associations with functional and social outcomes in individuals with psychosis [31, 32].

Women with psychosis were five times more likely to move from supported to independent living than men. In general, sex and gender differences result in varying clinical and social experiences that could explain this finding. First, men with psychosis are often diagnosed earlier (18–24 years) than women (up to four years later) [33]. As women are often diagnosed in their late 20 s, they may already have an established social network that can provide them with support in transitioning towards living in their own homes. Such support has been identified as a crucial factor in facilitating the successful transition towards independence [5]. Second, women with psychosis have higher levels of insight into their symptoms [34] with poor insight correlated with lower treatment adherence and therapeutic alliance [35]. As such, women tend to be more likely to adhere to and respond to regimens and programs, which could increase their chances of moving towards more independent living. Finally, gender roles and societal expectations may also influence this difference, though these factors are culturally dependent and can vary across different societies and countries.

Furthermore, the *course* of positive symptoms rather than their *baseline severity* was predictive of the transition from supported to independent living. Our findings are in line with existing literature in that positive symptoms measured at a single time point were not associated with a more independent living situation [10, 11]. In current research, positive symptoms are often only measured once at baseline, while health care professionals look at the long-term development of symptoms in clinical practice. Further investigation is warranted as to whether the trajectory of positive symptoms is a better predictor of prospective functioning and independent living than a single measurement. Furthermore, this finding underscores the substantial burden associated with treatment-resistant psychosis in terms of functional limitations and higher health care costs [36].

### Implications for clinical practice and research

So far, few studies have investigated interventions for individuals with severe mental illness in supported housing [37]. Our results, therefore, inform clinical practice and research about the factors that could be addressed in such interventions. First, cognitive remediation and compensatory cognitive interventions, that can improve cognitive and functional outcomes in individuals with psychosis [38, 39], might be offered to individuals with cognitive impairments to increase the probability of living independently in the future. Second, researchers should investigate different treatment regimens that might improve outcomes for men with psychosis living in supported housing, for example, the addition of more psychosocial interventions and social support. Third, it is crucial to focus on improving treatment for individuals with treatment-resistant psychosis in clinical practice and research. Finally, it is crucial for society to provide support to researchers in the development of interventions aimed at assisting individuals who face challenges in transitioning out of supported housing situations. By doing so, the long-term costs associated with a large number of individuals relying on supported living can be reduced.

### Strengths and limitations

The major strengths of this study are its large sample size compared to prior studies on this topic, the method, which looks specifically at predictors of transition instead of associations of variables, and the consideration of clinical variability in symptoms using trajectories. However, the following limitations should be considered when interpreting the results. First, we dichotomized outcomes into *supported* and *independent* living for statistical reasons. In turn, there are several questions left unanswered about predictors of moving to or away from one specific housing state (i.e., whether predictors differ between moving towards living alone or towards living with a partner). Second, more severely ill or lower functioning individuals may be underrepresented because of two reasons: (1) selection bias of the GROUP sample, and (2) missing information about the housing state resulting in the exclusion of participants from Markov Chain modeling and the association analysis. Thirdly, our recommendations primarily focus on interventions to improve cognitive functioning and address treatment-resistant psychosis. However, we acknowledge the importance of other factors and interventions that promote independent living. While our study lacks a measure of daily living skills, prior research indicates that interventions like shopping skills training, cognitive behavioral training, social skills training, and psychoeducation can improve social and independent functioning [40], hence, possibly increasing the chances of moving from supported to independent living. Various practical and societal factors (e.g., supportive social network, available societal support, financial constraints, housing availability, political context, stigma), both directly and indirectly related to psychosis, can also influence the possibilities of living independently. Future studies should consider these aspects to understand barriers and facilitators for individuals with psychosis aiming for living independently. Finally, as structure of psychiatric care facilities differs between countries while culture might influence definition and connotation of living conditions, our research is primarily applicable in countries with similar systems and culture. Thus, living independently should be seen as a part of normal adulthood while independent and supported housing should be clearly distinguishable.

## Conclusions

This study shows that, in this sample, most individuals with non-affective psychosis living in sheltered housing or with their parents did not move towards independent living within a follow-up period of six years. Older men with lower cognitive functioning, especially in executive function, and a more severe course of positive symptoms were at risk of not moving into independent accommodation. Future research should investigate the effectiveness of cognitive interventions for individuals living in sheltered housing and with their parents. Furthermore, such research should pay particular attention to the variability of clinical symptoms over time when assessing predictors of functional outcomes and should focus on improving approaches to treatment-resistant psychosis. Finally, sex differences should also be considered in future research and treatment decisions.

## Supplementary Information

Below is the link to the electronic supplementary material.

Supplementary file1 (PDF 225 KB)

## Data Availability

The GROUP study has been concluded in 2016. Researchers must submit the request for data access to the study board, which will arrange the access to data to investigators once the data sharing agreement (DTA) is approved by the legal department of Maastricht University Medical Center. Data will be shared following approval of the signed DTA, and through secure data transfer stream. Investigators will support the data sharing once they are requested. Participants’ identity must be kept anonymous, while the rest of the variables alongside a data dictionary will be made available. The study protocol has been published including a statistical analysis plan, and the informed consent form is available.

## References

[1] Hudson CG (2016) A model of deinstitutionalization of psychiatric care across 161 nations: 2001–2014. Int J Ment Health 45:135–153. 10.1080/00207411.2016.1167489

[2] Jose D, Ramachandra LK et al (2015) Consumer perspectives on the concept of recovery in schizophrenia: a systematic review. Asian J Psychiatr 14:13–18. 10.1016/J.AJP.2015.01.006 25703654 10.1016/j.ajp.2015.01.006

[3] Fakhoury WKH, Priebe S, Quraishi M (2005) Goals of new long-stay patients in supported housing: a UK study. Int J Soc Psychiatry 51:45–54. 10.1177/0020764005053273 15864974 10.1177/0020764005053273

[4] Browne G, Courtney M (2005) Housing, social support and people with schizophrenia: a grounded theory study. Issues Ment Health Nurs 26:311–326. 10.1080/01612840590915694 16020049 10.1080/01612840590915694

[5] Fossey E, Harvey C, McDermott F (2019) Housing and support narratives of people experiencing mental health issues: making my place, my home. Front Psychiatry. 10.3389/FPSYT.2019.00939 31998158 10.3389/fpsyt.2019.00939 PMC6966198

[6] Santesteban-Echarri O, Paino M, Rice S et al (2017) Predictors of functional recovery in first-episode psychosis: a systematic review and meta-analysis of longitudinal studies. Clin Psychol Rev 58:59–75. 10.1016/J.CPR.2017.09.007 29042139 10.1016/j.cpr.2017.09.007

[7] Brekke J, Kay DD, Lee KS, Green MF (2005) Biosocial pathways to functional outcome in schizophrenia. Schizophr Res 80:213–225. 10.1016/j.schres.2005.07.008 16137859 10.1016/j.schres.2005.07.008

[8] De Girolamo G, Candini V, Buizza C et al (2014) Is psychiatric residential facility discharge possible and predictable? A multivariate analytical approach applied to a prospective study in Italy. Soc Psychiatry Psychiatr Epidemiol 49:157–167. 10.1007/s00127-013-0705-z 23712514 10.1007/s00127-013-0705-z

[9] Dickerson FB, Ringel N, Parente F (1999) Predictors of residential independence among outpatients with schizophrenia. Psychiatr Serv 50:515–519. 10.1176/ps.50.4.515 10211733 10.1176/ps.50.4.515

[10] Auslander LA, Lindamer LL, Delapena J et al (2001) A comparison of community-dwelling older schizophrenia patients by residential status. Acta Psychiatr Scand 103:380–386. 10.1034/j.1600-0447.2001.00262.x 11380308 10.1034/j.1600-0447.2001.00262.x

[11] Palmer BW, Heaton RK, Gladsjo JA et al (2002) Heterogeneity in functional status among older outpatients with schizophrenia: employment history, living situation, and driving. Schizophr Res 55:205–215. 10.1016/S0920-9964(01)00218-3 12048144 10.1016/s0920-9964(01)00218-3

[12] Hofer A, Baumgartner S, Bodner T et al (2005) Patient outcomes in schizophrenia II: the impact of cognition. Eur Psychiatry 20:395–402. 10.1016/j.eurpsy.2005.02.006 16171654 10.1016/j.eurpsy.2005.02.006

[13] Levitt JJ, Shenton ME, McCarley RW et al (1994) Premorbid adjustment in schizophrenia: implications for psychosocial and ventricular pathology. Schizophr Res 12:159–168. 10.1016/0920-9964(94)90073-6 8043526 10.1016/0920-9964(94)90073-6

[14] Palmer BW, Dawes SE, Heaton RK (2009) What do we know about neuropsychological aspects of schizophrenia? Neuropsychol Rev 19:365–384. 10.1007/s11065-009-9109-y 19639412 10.1007/s11065-009-9109-y PMC2745531

[15] Habtewold TD, Rodijk LH, Liemburg EJ et al (2020) A systematic review and narrative synthesis of data-driven studies in schizophrenia symptoms and cognitive deficits. Transl Psychiatry 10:1–24. 10.1038/s41398-020-00919-x 32694510 10.1038/s41398-020-00919-x PMC7374614

[16] Korver N, Quee PJ, Boos HBM et al (2012) Genetic Risk and Outcome of Psychosis (GROUP), A multi site longitudinal cohort study focused on gene-environment interaction: objectives, sample characteristics, recruitment and assessment methods. Int J Methods Psychiatr Res 21:205–221. 10.1002/mpr.1352 22419500 10.1002/mpr.1352 PMC6878383

[17] Meijer J, Simons CJP, Quee PJ et al (2012) Cognitive alterations in patients with non-affective psychotic disorder and their unaffected siblings and parents. Acta Psychiatr Scand 125:66–76. 10.1111/j.1600-0447.2011.01777.x 22013907 10.1111/j.1600-0447.2011.01777.x

[18] Kay SR, Fiszbein A, Opler LA (1987) The positive and negative syndrome scale (PANSS) for schizophrenia. Schizophr Bull 13:261–276. 10.1093/schbul/13.2.261 3616518 10.1093/schbul/13.2.261

[19] Van Der Meer L, Kaiser S, Castelein S (2021) Negative symptoms in schizophrenia: reconsidering evidence and focus in clinical trials. Br J Psychiatry 219:359–360. 10.1192/bjp.2021.66 35048858 10.1192/bjp.2021.66

[20] Stiekema APM, Liemburg EJ, Van Der Meer L et al (2016) Confirmatory factor analysis and differential relationships of the two subdomains of negative symptoms in chronically ill psychotic patients. PLoS ONE. 10.1371/journal.pone.0149785 26895203 10.1371/journal.pone.0149785 PMC4760738

[21] Cannon-Spoor HE, Potkin SG, Jed Wyatt R (1982) Measurement of premorbid adjustment in chronic schizophrenia. Schizophr Bull 8:470–480. 10.1093/schbul/8.3.470 7134891 10.1093/schbul/8.3.470

[22] Slade M, Beck A, Bindman J et al (1999) Routine clinical outcome measures for patients with severe mental illness: CANSAS and HoNOS. Br J Psychiatry 174:404–408. 10.1192/BJP.174.5.404 10616605 10.1192/bjp.174.5.404

[23] Islam MA, Habtewold TD, van Es FD et al (2018) Long-term cognitive trajectories and heterogeneity in patients with schizophrenia and their unaffected siblings. Acta Psychiatr Scand 138:591–604. 10.1111/acps.12961 30242827 10.1111/acps.12961 PMC6220939

[24] Habtewold TD, Tiles-Sar N, Liemburg EJ et al (2023) Six-year trajectories and associated factors of positive and negative symptoms in schizophrenia patients, siblings, and controls: Genetic Risk and Outcome of Psychosis (GROUP) study. Sci Rep. 13(1):9391. 10.1038/s41598-023-36235-9 37296301 10.1038/s41598-023-36235-9 PMC10256804

[25] Tiles-Sar N, Habtewold TD, Liemburg EJ et al (2023) Understanding lifelong factors and prediction models of social functioning after psychosis onset using the large-scale GROUP Cohort study. Schizophr Bull. 10.1093/schbul/sbad046 37104875 10.1093/schbul/sbad046 PMC10686366

[26] Jackson CH (2011) Multi-state models for panel data: The MSM package for R. J Stat Softw 38:1–28. 10.18637/jss.v038.i08

[27] Core Development Team R (2020) A language and environment for statistical computing. R foundation for statistical computing 2: https://www.R-project.org

[28] Castelein S, Timmerman ME, PHAMOUS investigators, van der Gaag M, Visser E (2021) Clinical, societal and personal recovery in schizophrenia spectrum disorders across time: states and annual transitions. Br J Psychiatry. 219(1):401–408. 10.1192/bjp.2021.48 35048855 10.1192/bjp.2021.48 PMC8529640

[29] Barnes S, Carson J, Gournay K (2022) Enhanced supported living for people with severe and persistent mental health problems: a qualitative investigation. Health Soc Care Commun 30(6):e4293–e4302. 10.1111/hsc.13822 10.1111/hsc.13822 PMC10084301 35524392

[30] Bodén R, Abrahamsson T, Holm G, Borg J (2014) Psychomotor and cognitive deficits as predictors of 5-year outcome in first-episode schizophrenia. Nord J Psychiatry 68:282–288. 10.3109/08039488.2013.830771 24050122 10.3109/08039488.2013.830771

[31] Tan BL (2009) Profile of cognitive problems in schizophrenia and implications for vocational functioning. Aust Occup Ther J 56:220–228. 10.1111/j.1440-1630.2008.00759.x 20854522 10.1111/j.1440-1630.2008.00759.x

[32] Fett AKJ, Viechtbauer W, de Dominguez MG et al (2011) The relationship between neurocognition and social cognition with functional outcomes in schizophrenia: a meta-analysis. Neurosci Biobehav Rev 35:573–588. 10.1016/j.neubiorev.2010.07.001 20620163 10.1016/j.neubiorev.2010.07.001

[33] Gogos A, Sbisa AM, Sun J et al (2015) A role for estrogen in schizophrenia: clinical and preclinical findings. Int J Endocrinol. 10.1155/2015/615356 26491441 10.1155/2015/615356 PMC4600562

[34] Comacchio C, Lasalvia A, Bonetto C et al (2020) Gender and 5-years course of psychosis patients: focus on clinical and social variables. Arch Women’s Mental Health 23:63–70. 10.1007/s00737-019-0945-3 10.1007/s00737-019-0945-3 30719573

[35] Lysaker PH, Pattison ML, Leonhardt BL et al (2018) Insight in schizophrenia spectrum disorders: relationship with behavior, mood and perceived quality of life, underlying causes and emerging treatments. World Psychiatry 17:12–23. 10.1002/wps.20508 29352540 10.1002/wps.20508 PMC5775127

[36] Kane JM, Agid O, Baldwin ML et al (2019) Clinical guidance on the identification and management of treatment-resistant schizophrenia. J Clin Psychiatry. 10.4088/JCP.18COM12123 30840788 10.4088/JCP.18com12123

[37] Bitter N, Roeg D, van Nieuwenhuizen C, van Weeghel J (2020) Recovery in supported accommodations: a scoping review and synthesis of interventions for people with severe mental illness. Commun Ment Health J 56:1053–1076. 10.1007/s10597-020-00561-3 10.1007/s10597-020-00561-3 PMC7289772 32016620

[38] Vita A, Barlati S, Ceraso A et al (2021) Effectiveness, core elements, and moderators of response of cognitive remediation for schizophrenia: a systematic review and meta-analysis of randomized clinical trials. JAMA Psychiat 78:848–858. 10.1001/jamapsychiatry.2021.0620 10.1001/jamapsychiatry.2021.0620 PMC8058696 33877289

[39] Stiekema APM, van Dam MT, Bruggeman R et al (2020) Facilitating recovery of daily functioning in people with a severe mental illness who need longer-term intensive psychiatric services: results from a cluster randomized controlled trial on cognitive adaptation training delivered by nurses. Schizophr Bull 46:1259–1268. 10.1093/schbul/sbz135 32144418 10.1093/schbul/sbz135 PMC7505172

[40] John A, Gandhi S, Prasad MK, Manjula M (2022) Effectiveness of IADL interventions to improve functioning in persons with Schizophrenia: a systematic review. Int J Soc Psychiatry 68(3):500–513. 10.1177/00207640211060696 34802260 10.1177/00207640211060696

