# Supplementary material for: Moving from supported to independent living: what are the barriers and facilitators for individuals with psychosis?
Source: Soc Psychiatry Psychiatr Epidemiol. 2024 Jan 8;59(7):1243–54. doi: 10.1007/s00127-023-02586-x (PMC11636980; doi:10.1007/s00127-023-02586-x)

Social Psychiatry and Epidemiologic Psychiatry

Supplementary Material

Moving from supported to independent living: what are the barriers and facilitators for individuals with psychosis?

Anika Poppe, M.Sc.[1,2]*; a.poppe@rug.nl (corresponding author); Natalia Tiles-Sar, M.Sc.[3,4]*; Stefan R.A Konings, M.Sc.[4,5]; Tesfa Dejenie Habtewold, Ph.D.[3];

GROUP Investigators, Richard Bruggeman, Ph.D.[4]; Behrooz Z. Alizadeh, Ph.D.[3]; Lisette van der Meer, Ph.D.[1,2]

Affiliations:
[1] University of Groningen, Department of Clinical and Developmental Neuropsychology, Groningen, The Netherlands. [2] Lentis Psychiatric Institute, Zuidlaren, Department of Rehabilitation, The Netherlands. [3] University Medical Center Groningen, University of Groningen, Department of Epidemiology, Groningen, The Netherlands. [4] University Medical Center Groningen, University Center for Psychiatry, University of Groningen, Department of Psychiatry, Rob Giel Research Center, Groningen, The Netherlands. [5] University Medical Center Groningen, University of Groningen, Department of Psychiatry, Interdisciplinary Center Psychopathology and Emotion Regulation (ICPE), Groningen, The Netherlands

GROUP Investigators:
Behrooz Z. Alizadeh, Ph.D., Therese van Amelsvoort, Ph.D., Agna A. Bartels-Velthuis, Ph.D., Richard Bruggeman, Ph.D., Wiepke Cahn, Ph.D. Lieuwe de Haan, Ph.D., Frederike Schirmbeck, Ph.D., Claudia J.P. Simons, Ph.D., Jim van Os, Ph.D.

Table S1 Descriptive characteristics of the overall sample, completers and those lost to follow-up at each wave of assessment

			3-year follow-up			6-year follow-up		
	All individuals (n= 1119)		Completers (n= 744)	Lost to follow-up (n= 375)	Comparison	Completers (n= 599)	Lost to follow-up (n= 520)	Comparison
	No. missing (%)	% / mean (SD)	% / mean (SD)	% / mean (SD)	P-value[a]	% / mean (SD)	% / mean (SD)	P-value[a]
**Demographical characteristics**								
Age at the baseline	4 (0.4%)	27.6 (7.98)	27.33 (7.27)	28.13 (9.22)	.115	27.41 (7.37)	27.82 (8.63)	.387
Sex: male	none	76.1%	76.2%	76.0%	.938	74.8%	77.7%	.256
Ethnicity: Caucasian	74 (6.6%)	79.4%	83.8%	69.8%	<.001	84.5%	73.1%	<.001
Living conditions								
Single	105 (9.4%)	33.4%	34.0%	31.8%	.761	34.1%	32.3%	.183
With parent(s)		39.6%	39.0%	41.4%		40.3%	38.6%	
With a partner/family		10.5%	10.8%	9.6%		10.8%	9.8%	
Sheltered living		9.8%	10.0%	9.2%		9.5%	10.1%	
Other		6.7%	6.2%	8.0%		5.2%	9.1%	
Employment								
None	192 (17%)	44.7%	41.9%	50.5%	.028	40.3%	50.0%	.010
Full-time		28.7%	31.0%	23.7%		30.1%	26.9%	
Part-time		26.6%	27.1%	25.8%		29.5%	23.1%	
Marital status								
Not married	41 (3.7%)	88.1%	87.4%	89.6%	.075	87.3%	89.1%	.368
Married/living together		9.1%	10.2%	6.7%		10.2%	7.8%	
Divorced		2.8%	2.3%	3.8%		2.5%	3.1%	
**Clinical characteristics – status at the baseline**								
Number of psychotic episodes	48 (4.3%)	1.75 (1.16)	1.74 (1.12)	1.76 (1.24)	.823	1.76 (1.11)	1.73 (1.21)	.644

	All individuals (n= 1119)		3-year follow-up: Completers (n= 744)	3-year follow-up: Lost to follow-up (n= 375)	3-year follow-up: Comparison	6-year follow-up: Completers (n= 599)	6-year follow-up: Lost to follow-up (n= 520)	6-year follow-up: Comparison
	No. missing (%)	% / mean (SD)	% / mean (SD)	% / mean (SD)	P-value[a]	% / mean (SD)	% / mean (SD)	P-value[a]
Age of onset, first psychosis	4 (0.4%)	23.07 (7.81)	22.40 (6.66)	24.40 (9.56)	<.001	22.53 (6.78)	23.70 (8.83)	.012
Duration of illness	104 (9.3%)	4.98 (4.46)	5.26 (4.54)	4.37 (4.25)	.003	5.17 (4.48)	4.74 (4.44)	.127
Medication use (chlorpromazine equivalent)	322 (28.7%)	460.82 (1134.11)	467.09 (1228.56)	446.29 (878.71)	.810	455.12 (1310.04)	468.15 (858.77)	.87
Unmet needs of social support	124 (11.1%)	3.24 (2.89)	2.95 (2.75)	3.87 (3.11)	<.001	2.89 (2.78)	3.67 (2.99)	<.001
**Substance abuse – status at the baseline**								
Alcohol (number of units per week)	26 (2.3%)	6.61 (12.05)	6.51 (10.71)	6.82 (14.44)	.693	6.72 (10.66)	6.48 (13.52)	.746
Cannabis (lifetime most intensive mode of use)								
None	25 (2.2%)	36.9%	38.1%	34.5%	.162	39.8%	33.6%	.057
Less than weekly		8.7%	9.6%	6.7%		9.5%	7.8%	
Weekly		10.9%	10.4%	11.8%		10.8%	10.9%	
Daily		43.5%	41.8%	47.1%		39.9%	47.7%	
**Trajectories**								
Premorbid adjustment, original groups								
Normal, slow decrease	none	13.9%	15.2%	11.2%	.019	16.5%	10.8%	.070
Mild, slow decrease	none	51.9%	48.4%	58.9%		48.4%	56.0%	
Normal, rapid decrease	none	7.1%	7.9%	5.3%		7.0%	7.1%	
Mild, rapid decrease	none	4.1%	4.6%	3.2%		4.2%	4.0%	
Moderate, slow decrease	none	18.4%	18.7%	17.9%		19.2%	17.5%	
Severe, slow decrease	none	4.6%	5.2%	3.5%		4.7%	4.6%	
Premorbid adjustment, combined groups								
Normal to mild, slow decrease	none	65.8%	63.6%	70.1%	.053	64.9%	66.7%	.775

	All individuals (n= 1119)		3-year follow-up: Completers (n= 744)	3-year follow-up: Lost to follow-up (n= 375)	3-year follow-up: Comparison	6-year follow-up: Completers (n= 599)	6-year follow-up: Lost to follow-up (n= 520)	6-year follow-up: Comparison
	No. missing (%)	% / mean (SD)	% / mean (SD)	% / mean (SD)	P-value[a]	% / mean (SD)	% / mean (SD)	P-value[a]
Normal to mild, rapid decrease	none	11.2%	12.5%	8.5%		11.2%	11.2%	
Moderate to severe, slow decrease	none	23.1%	23.9%	21.3%		23.9%	22.1%	
Cognitive deficit, original groups								
High	none	10.1%	12.5%	5.3%	<.001	13.5%	6.2%	<.001
Normal	none	31.5%	35.5%	23.7%		38.2%	23.8%	
Mild	none	41.6%	37.1%	50.7%		33.6%	51.0%	
Moderate	none	14.4%	13.0%	17.1%		13.5%	15.4%	
Severe	none	2.3%	1.9%	3.2%		1.2%	3.7%	
Cognitive deficit, combined groups								
High to normal	none	41.6%	48.0%	29.1%	<.001	51.8%	30.0%	<.001
Mild	none	41.6%	37.1%	50.7%		33.6%	51.0%	
Moderate to severe	none	16.7%	14.9%	20.3%		14.7%	19.0%	
Negative symptoms								
Low	none	74.0%	74.6%	72.8%	.038	75.6%	72.1%	.189
High, decreasing severity	none	14.3%	12.6%	17.6%		12.5%	16.3%	
High, increasing severity	none	11.7%	12.8%	9.6%		11.9%	11.5%	
Positive symptoms								
Low	none	70.4%	71.6%	68.0%	.271	72.3%	68.3%	.110
Moderate	none	21.2%	20.8%	21.9%		20.9%	21.5%	
Severe	none	8.4%	7.5%	10.1%		6.8%	10.2%	

[a]The difference between completers and those lost at 3-year follow-up and 6-year follow-up was assessed with chi-square goodness of fit test for categorical variables and two-sample t-test for continuous variables.

Fig. S1 Frequencies and transitions probabilities of individuals in each housing state

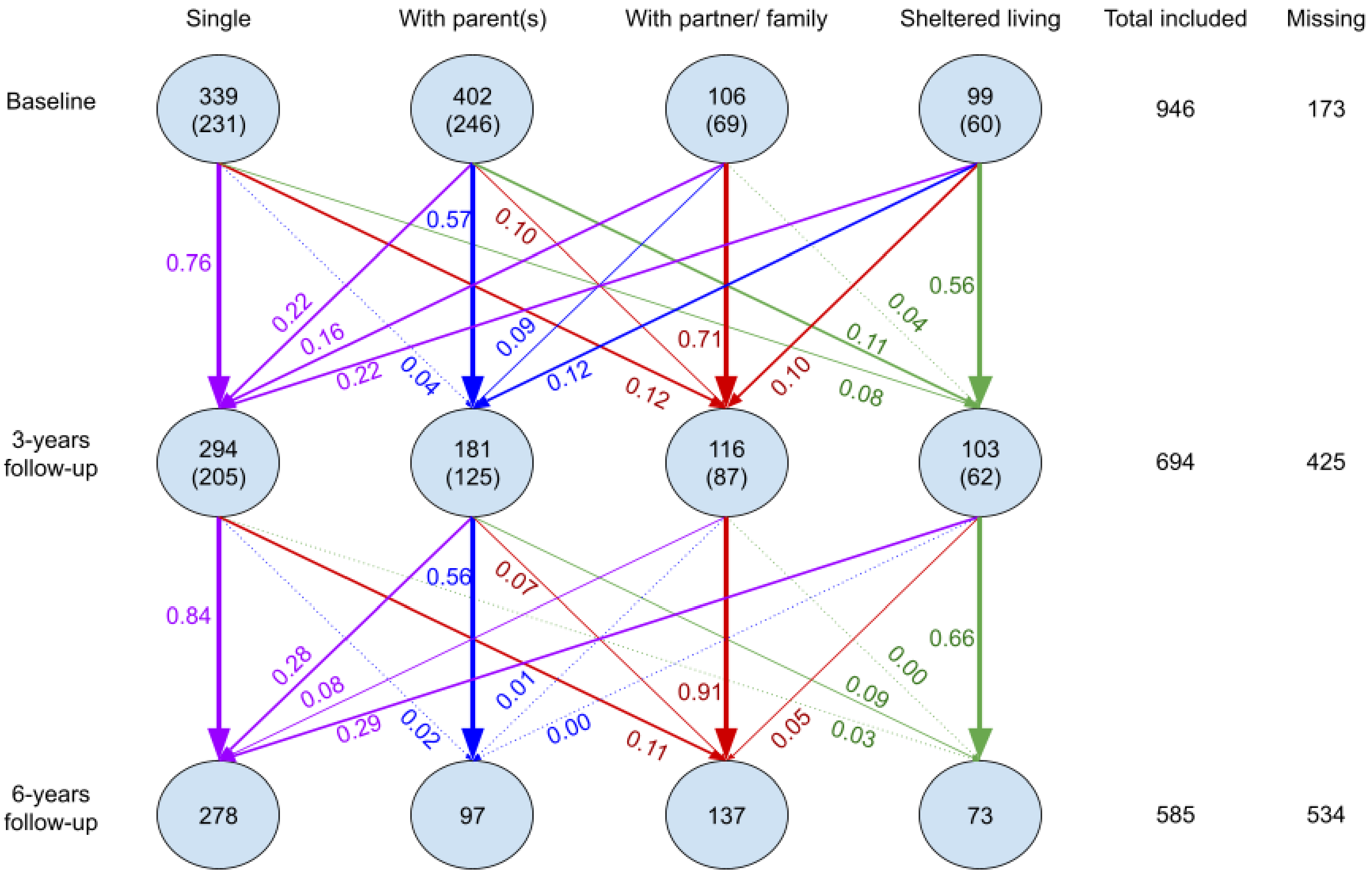

Supplement: Supplementary file 1 — Supplementary file1 (PDF 225 KB) [file 127_2023_2586_MOESM1_ESM.pdf]
